# UniDrug-Target: A Computational Tool to Identify Unique Drug Targets in Pathogenic Bacteria

**DOI:** 10.1371/journal.pone.0032833

**Published:** 2012-03-14

**Authors:** Sree Krishna Chanumolu, Chittaranjan Rout, Rajinder S. Chauhan

**Affiliations:** Department of Biotechnology and Bioinformatics, Jaypee University of Information Technology, Waknaghat, Solan, Himachal Pradesh, India; Semmelweis University, Hungary

## Abstract

**Background:**

Targeting conserved proteins of bacteria through antibacterial medications has resulted in both the development of resistant strains and changes to human health by destroying beneficial microbes which eventually become breeding grounds for the evolution of resistances. Despite the availability of more than 800 genomes sequences, 430 pathways, 4743 enzymes, 9257 metabolic reactions and protein (three-dimensional) 3D structures in bacteria, no pathogen-specific computational drug target identification tool has been developed.

**Methods:**

A web server, UniDrug-Target, which combines bacterial biological information and computational methods to stringently identify pathogen-specific proteins as drug targets, has been designed. Besides predicting pathogen-specific proteins essentiality, chokepoint property, etc., three new algorithms were developed and implemented by using protein sequences, domains, structures, and metabolic reactions for construction of partial metabolic networks (PMNs), determination of conservation in critical residues, and variation analysis of residues forming similar cavities in proteins sequences. First, PMNs are constructed to determine the extent of disturbances in metabolite production by targeting a protein as drug target. Conservation of pathogen-specific protein's critical residues involved in cavity formation and biological function determined at domain-level with low-matching sequences. Last, variation analysis of residues forming similar cavities in proteins sequences from pathogenic versus non-pathogenic bacteria and humans is performed.

**Results:**

The server is capable of predicting drug targets for any sequenced pathogenic bacteria having fasta sequences and annotated information. The utility of UniDrug-Target server was demonstrated for *Mycobacterium tuberculosis* (H37Rv). The UniDrug-Target identified 265 *mycobacteria* pathogen-specific proteins, including 17 essential proteins which can be potential drug targets.

**Conclusions/Significance:**

UniDrug-Target is expected to accelerate pathogen-specific drug targets identification which will increase their success and durability as drugs developed against them have less chance to develop resistances and adverse impact on environment. The server is freely available at http://117.211.115.67/UDT/main.html. The standalone application (source codes) is available at http://www.bioinformatics.org/ftp/pub/bioinfojuit/UDT.rar.

## Introduction

Most drugs exert therapeutic effects by binding and regulating the activity of a particular protein, set of proteins or nucleic acid targets in the pathogenic microbes. The identification and validation of such targets compose an important step in drug discovery process [1], [2]. Despite beneficial impacts of antibacterials in curing infectious diseases, these molecules reach the environment through excretion or accidental leakages. The molecules then kill beneficial microbes (agricultural, industrial or of other importance) and non-pathogenic bacteria, including probiotic bacteria which are indispensable for survival of humans and animals. These adverse effects arise due to antibacterial drugs targeting common proteins in bacteria without discriminating between pathogenic and non-pathogenic. Studies have shown that up to 90 percent of the antibiotics used are excreted out and able to reach water reservoirs without metabolizing. These antibiotics reside in water reservoirs even after ‘water treatment’ designed to remove waste and toxic materials. These water soluble antibiotics, due to prolonged interactions with bacteria, stimulate bacterial metabolism leading to the selection and maintenance of antibiotic resistance genes which were acquired through horizontal gene transfer [3]. Use of antibacterials also produce side-effects and disturbs probiotic host microbiota resulting in not only gastrointestinal tract problems but also increases susceptibility of human to enteric pathogens [4]. Since evolutionary conservation of some receptors and metabolic pathways has been preserved in plants and bacteria, antibacterial (antibiotics) drugs also target protein receptors in plants and disturb various processes such as chloroplast replication by fluoroquinolones; transcription and translation processes by tetracyclines macrolides, lincosamides, P-aminoglycosides, and pleuromutilins; metabolic pathways for example folate and fatty acid biosyntheses through sulfonamides and triclosan, respectively [5].

The use of antibacterial drugs targeting proteins conserved among bacteria is also an important causative factor for the development of drug resistance. Fluoroquinolone-resistance was observed in new tuberculosis (TB) patients who were administered with a commonly used antibiotic, fluoroquinolones (more than 10 days), for the treatment of sinusitis, pneumonia, urinary tract infection, etc. prior to their diagnosis of TB. The chance and extent of resistance to TB was proportional to period of consumption of fluoroquinolones [6]. When people infected with inactive *Mycobacterium tuberculosis* were administered fluoroquinolones for the treatment of sinusitis, pneumonia, etc., since these antibiotics target DNA gyrase (topoisomerase II) and topoisomerase IV, the inactive *M. tuberculosis* evolved to fluoquinolones-resistant strains. The drugs intended to kill *Streptococcus pneumoniae* also targeted *M. tuberculosis* as the targets were common in both the pathogens [6]. A potential link was established between the use of fluoroquinolones for the treatment of bacterial infections (other than *Clostridium difficile*) and the development of more virulent strains of *C. difficile* infection in hospitalized patients arising due to common drug target [7].

Various drug target identification techniques [8], [9] have been developed by analyzing disease relevance, functional roles, expression profiles and loss-of-function genetics between normal and disease states [10]–[14]. Most of the computational methods are based on detection of sequence and functional similarity to known targets and drug-binding domain families affiliation [15], [16]. Structural analysis parameters that describe polar and apolar surface areas, surface complexity, and pocket dimensions have also been used to identify drug targets [17]. Most of the earlier databases, servers and programs such as PDTD database [18], TarFisDock [19] and InvDock (http://bidd.nus.edu.sg/group/softwares/invdock.htm) required 3D structure of protein to determine its drug target potential. These methods were less effective in finding targets that exhibit no or low homology to proteins with available 3D structures, known targets and proteins involved in disease process. Support vector machine (SVM) based methods have been developed from amino acid sequence-derived properties of known and research targets to predict druggable proteins from bacterial proteome sequences [20], [21]. These SVM methods are having the problems of overprediction. These methods are expected to predict proteins highly similar to human and nonpathogenic sequences as human and conserved proteins from bacteria(both pathogenic and non-pathogenic) were used for training and development of SVM. Moreover, none of these computational methods are available online to the users for identifying specific targets in any pathogenic organism despite the availability of numerous genome sequences and colossal amount of biological information on bacteria. The earlier computational methods neither addressed adverse effects of antibiotics on beneficial microbes in the environment nor the development of resistant strains. Also, those methods did not provide any information about the biological function of a target which is a significant parameter. The development of a freely accessible pathogen-specific computational drug target identification method supported through a server would enable decision processes considering several parameters at a time on one platform. The server would also ease the tedious job to analyze and correlate information from multiple datasets and applications individually for each protein which otherwise has been a stumbling block for researchers in choosing the best target(s) in an automated manner.

A web server, named as UniDrug-Target, which provides potential bacterial pathogenic-specific drug targets from given proteome(s) sequences, has been developed. The server compares proteome sequences of pathogenic strain(s) against sequences of nonpathogenic and beneficial strains of bacteria as well as human proteomes to identify unique proteins in the former. Information about the biological roles of unique proteins in pathogenesis or protection from the host is also provided. The importance of a unique protein as a potential drug target is determined on the basis of its functional importance, i.e. essentiality in cell survival, chokepoint property (enzyme catalyzes a reaction in which either a substrate/product is uniquely consumed/produced), function and domain information, involvement in different pathways, and disturbances in metabolite productions by targeting pathogen-specific enzyme by a drug. The essential genes of an organism constitute its minimal gene set which would be sufficient to sustain a functioning cellular life form under most favourable conditions [22]. Since end metabolite production within a pathway depends upon sequential completion of all reactions in a pathway and supporting reactions from other pathways, therefore, inhibition of any enzyme in the upstream regulating reactions would lead to cessation of its production and might cause disturbances in various dependent metabolic pathways which use the given end metabolite as a substrate. It is expected that the inhibition of a unique chokepoint enzyme would lead to either accumulation of the unique substrate (potentially toxic to the cell) or starvation of the unique product (potentially crippling essential cell functions) as no other shunt path would be available to execute the reaction. Chokepoints are, therefore, considered as good drug targets and more than 85% of the reported drug targets of *Plasmodium falciparum* are chokepoints [23]. The chokepoints which produce or consume essential metabolites have been proposed as drug targets and the analogs for essential metabolites binding to the chokepoints were proposed as drugs [24]. As metabolic processes work in coordination with one another in a living system forming metabolic networks which share or transfer the metabolites within it, disturbance in one of the elements in a metabolic network (enzyme/pathway) would result in metabolite imbalances which in turn will have serious repercussions on the pathogen's survival. The server, therefore, provides information related to pathways getting disturbed by inhibiting or disrupting a given unique enzyme. The server also provides information about the residues conserved at the domain level and 3D spatial arrangements of residues at the cavity site with uniqueness in composition of residues forming cavities in the pathogenic organisms compared to the nonpathogenic and human sequences. The server predicts unique cavity information for potential targets so that the drugs designed against those cavities would not bind to beneficial/nonpathogenic organisms. The utility of web server has been demonstrated in *M. tuberculosis*.

## Materials and Methods

### Data Collection and Generation

The computational methods used to collect and generate data are described here under:

#### Proteomes information

Proteomes for the construction of web server were collected from the NCBI (ftp://ftp.ncbi.nih.gov/genomes/Bacteria/all.faa.tar.gz). Classification of bacterial species into pathogenic and non-pathogenic was done by using genome project information given for each organism by the NCBI genome browser (http://www.ncbi.nlm.nih.gov/sites/genome). GI(NCBI GenBank Id) No. and corresponding synonymous names were collected from all.ptt file in the NCBI (ftp://ftp.ncbi.nih.gov/genomes/Bacteria/all.ptt.tar.gz) and annotation information was used for clustering proteins into their respective KO(KEGG orthologous) groups. Sequences of human proteome were collected from the KEGG ftp(http://www.kegg.jp/kegg/download/).

#### Enzymes, Reactions, Pathways and Metabolites Data

Pathways, reactions, compounds and proteomes of all bacteria were collected from the KEGG FTP (ftp://ftp.genome.jp/pub/kegg). Enzymes in bacterial proteomes were classified according to their EC No.(Enzyme Classification Number)(ftp://ftp.genome.jp/pub/kegg/genes/fasta/genes.pep). Reaction data source (ftp://ftp.genome.jp/pub/kegg/ligand/reaction/reaction) was used to identify enzymes and metabolites involved in a reaction, and development of metabolic networks. Compound (metabolite) file was collected from the KEGG(ftp://ftp.genome.jp/pub/kegg/ligand/compound). Pathways represented in module files at the KEGG (ftp://ftp.genome.jp/pub/kegg/module/module) were used to collect reactions involved in a pathway and the order of reactions, and orthologous genes in different organisms involved in corresponding pathways. The whole data sets mentioned above were used for the development of bacterial partial metabolic networks (PMNs).

#### Essential Genes

The essential gene sequences were retrieved from database of essential genes, DEG 5.0 [25].

#### Chokepoints

Chokepoints reaction analysis was done through in-house developed Perl scripts for 9,257 metabolic reactions resulting in the identification of 2,754 and 2,957 reactions in all bacteria which uniquely consume and produce metabolites, respectively. Chokepoint enzymes and their corresponding ranks for 386 pathogenic and non-pathogenic organisms were taken from Rahman *et al.* [26], [27]. The ranks of chokepoint enzymes for individual organisms were stored in a flat file along with their synonymous names and GI information. These chokepoint sequences were stored in a database called as Chokepoint sequence database.

#### Domain information

Protein domains reported in the PFam (ftp://ftp.ddbj.nig.ac.jp/mirror_database/pfam/pfam-A.fasta) were used to identify low-matching conserved regions in unique pathogen protein sequences. These conserved regions were used to determine structure and functional variations at residue level among protein sequences from pathogen, non-pathogen and human at matching domain regions.

#### Active sites information

The 3D structures of proteins were collected from the protein data bank (PDB) (http://www.rcsb.org) and possible active sites and pockets were identified by using fpocket program [28]. For all structures, the residues identified by fpocket involved in the pocket formation along with chains were extracted using Perl scripts and were stored in flat files for their further use in determining functionally important residues in pockets.

#### Clustering of proteins in bacteria to KO groups

From the .ptt files (collected from ftp://ftp.ncbi.nih.gov/genomes/Bacteria/all.ptt.tar.gz), KEGG orthologous group names were retrieved for already annotated protein sequences. For sequences in which KEGG orthologous annotated information was not available, those were clustered to the best possible matching KO groups at sequence level so as to provide possible protein's biological role in bacteria.

The above mentioned data sets were collected or generated, reorganized and integrated as back end information in the UniDrug-Target server for automatic analysis.

### Algorithms

#### Identification of chokepoints

The metabolic reactions which either uniquely consume or produce metabolites were identified from the metabolic network information collected from KEGG FTP using perl scripts. Chokepoint enzymes were identified for 843 bacterial proteomes. Chokepoint reactions in bacterial metabolic networks were detected by identifying reactions uniquely consuming a substrate or producing a product in such a way that the reaction is not an end step in the entire metabolic network and connecting to multiple reactions present in various pathways. For these reactions, partial metabolic network (PMN) under its influence was provided in a hierarchical tree of pathways which showed the effect on metabolic network of bacteria by inhibiting the enzymes catalyzing respective chokepoint reactions.

#### Identification of interruption sites/points (Enzymes) in metabolic pathways and corresponding end metabolites production

Considering the reaction catalyzed by an enzyme as a starting point, the PMNs under the influence of the enzyme catalyzing set of reactions (directly or indirectly) were constructed. These were constructed in a hierarchical tree by using breadth first search method to traverse and add metabolic reactions. Paths leading to end metabolites for each enzyme reported in bacteria were determined based upon the assumption that the cell always maintains steady state for intermediate metabolites. The products of metabolic reactions are immediately consumed as substrates by the next subsequent metabolic reactions existing in the same pathways and only end metabolites are transferred from one pathway to the next in a metabolic network.

To identify pathways dependent (directly or indirectly) upon an enzyme from the identified unique proteins, the reaction catalyzed by the given enzyme was considered as a starting point. From this point, traversal of metabolic processes in the same pathway (mimicking the same order in which they execute in natural environments by considering product of previous reactions as substrate for the current reaction and end metabolic reaction products are not consumed by any reaction in the same pathway) was continued until the end metabolic reaction in the same pathway. Once the end metabolites were determined, the pathways in which the end metabolites were acting as substrates were identified, and the reactions initiated by these end-metabolites were considered as starting points for identifying next set of end metabolites in respective pathways. The pathways where first set of end metabolites were used as substrates determined the next level of pathways in the hierarchical tree dependent indirectly on a given enzyme. This process was iteratively repeated until no further metabolic pathway gets added into the network. The pathways were represented in a hierarchical tree in the order as they were identified and pathway repetition was not allowed. Perl scripts were written to construct all the PMNs in bacteria and pathways getting affected by inhibiting an enzyme (from unique proteins) using metabolic network information from the KEGG ftp location. The Perl scripts can be obtained from standalone distribution which currently containing information about 151 metabolic pathways and 9257 metablolic reactions. User can construct new reactions along with their asscociated enzymes and new pathways to construct dynamic pathway connectivity networks by including upcoming metabolic network information.

#### Identification of metabolic pathways disturbed by inhibition of dihydropyrimidine dehydrogenase (NADP+) of *M. tuberculosis*

**Figure 1:**
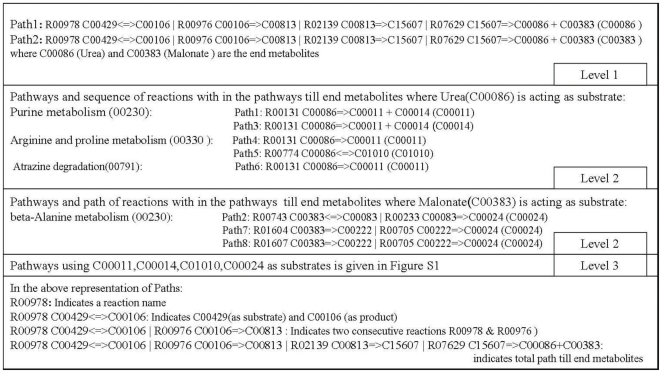
Metabolic paths identified until end metabolite synthesis in pyrimidine metabolism (00240) connected pathways starting from the reaction (R00978) catalyzed by the enzyme, dihydropyrimidine dehydrogenase (NADP+ [EC: 1.3.1.2], KEGG Orthologous Id: K00207).

The enzyme, dihydropyrimidine dehydrogenase in pyrimidine metabolism pathway, catalyzes the reaction, R00978 and consumption of its products by subsequent reactions results in the synthesis of two end metabolites: Urea (C00086) and Malonate (C00383) (Figure 1). The former metabolite can be synthesized by other pathways whereas the latter is produced only by this pathway; therefore, inhibition of this enzyme not only ceases the production of this metabolite but also will impact pathways which use this metabolite as a substrate. The pathways which use C00086 and C00383 as substrates are given in (Figure 1) (Level 2 section). Starting from the reaction(s) initiated by the end metabolites, C00086 and C00383, as substrates, the metabolic paths were traced to identify end metabolites in the respective connected pathways, and involvement of newly determined end metabolites as substrates in various other pathways and their end metabolites were determined. Connecting pathways using end metabolites iteratively (connecting pathways with the help of end metabolites) enabled to construct hierarchical tree of pathways by the order in which they appeared in the trace. Through this method, pyrimidine metabolism pathway has been connected with the pathways under its direct influence and subsequently end metabolites of the connected pathways under its direct influence were used to identify further connected pathways (Figure S1). This type of PMN analysis was done for all enzyme catalyzed reactions in entire bacterial networks and then mapped to 843 bacterial systems.

#### Identification of variations at functional sites to determine site specific uniqueness in pathogen protein sequences

Identification of low-matching protein sequences through similarity score and E-value of the BLAST is not always sufficient to determine the uniqueness of site in a drug target. As proteins preserve certain residues to carry out their functions in various organisms, the conserved residues within the low-matching regions of non-pathogenic and human sequences matching with the sequences of the pathogen were identified to determine whether functionality is preserved or not. For this purpose, low-matching unique protein sequences in the pathogenic organisms were matched with the Pfam domain family sequences to determine the presence of signature domains. The presence of domains in pathogen-specific proteins was checked in non-pathogenic and human proteomes. Low-matching domain regions of the pathogen-specific proteins were further analyzed for functionally important residues, which might be conserved in the low-matching proteins of non-pathogenic and human proteins. The matching domain sequences were further analyzed with the sequences of PDB database to map the domains on the available 3D structures and also to determine 3D orientations of the conserved residues within the domains. The conserved residues between the unique pathogen proteins and Pfam domain sequences involved in pocket formation were analyzed for their matching residues in non-pathogenic and human sequences to determine the amount of variability at the functional sites. The pathogen-specific unique sequences not showing the presence of a domain in the Pfam were matched as such with the available 3D structures to identify surface and pocket residues, if any, which may be involved in protein-protein and protein-substrate (drug) interactions. The protein cavites in which surface pocket residues were found in pathogen-specific sequences were also checked for residues in the non-pathogen and human proteins. As a result, the pathogen proteins variable at cavity sites with the non-pathogen and human proteins could be considered as better targetable sites.

Identification of critical residues that might be involved in cavity formation was done with the help of pocket information available from their respective matching PDB structures. Pathogen-specific protein sequences which were not having the exact match with the PDB database were analyzed through structures with low-matching sequences. The process undergoes as follows: pathogenic sequences were matched to the PDB sequences to collect matching 3D structure of the protein sequence (if high matching is not available then low-matching 3D structures were considered). All possible matching 3D structures information was used for guiding the user to identify critical residues and to determine their importance in cavity formation. These matching PDB sequences were then matched with the non-pathogen and human sequences. Matching residue positions were noted in all matching pathogenic and non-pathogenic sequences w.r.t. cavity forming residues for each matching 3D structure. The variability in residues forming the same cavity in pathogenic and non-pathogenic organisms indicates the uniqueness of the pathogenic-specific cavity and could be used to develop pathogen-specific drugs. Identification of critical residues in pathogen sequences involved in cavity formation has to be done by considering set of structures which cover the entire pathogen sequence at sequence level and different chains of the matching structures to cover the cavity forming residues from the unique cavity information generated by server, Unidrug-Target.

### Server Architecture and Implementation

**Figure 2:**
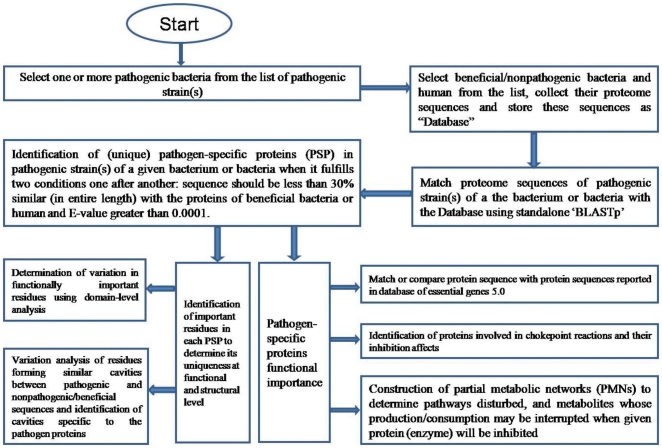
Flow diagram representing methodology implemented in UniDrug-Target server to identify potential pathogen-specific drug targets.

UniDrug-Target is an online web server developed to help user for easy identification of drug targets in pathogenic bacteria (Figure 2). User/client may refer to a tutorial available at the server's site to gain familiarity.

#### Client interface

Front-end was developed using HTML language which provides computational methodology in detail to identify pathogen-specific drug targets. To use this tool, the client/user needs to connect the server's home page available at URL location: http://117.211.115.67/UDT/main.html. User is required to click on ‘Click here to execute the server’ and then he/she can submit a new request to identify unique targets by selecting set of pathogenic and non-pathogenic organisms. After job submission, an html page link where output will be available is generated and user may bookmark it for later use to track on the status of a job. Initially status of the job will be ‘pending’. User can use the same address to know the status and whenever it changes to ‘Result’, he/she can click on the ‘Result’ button to retrieve final results. Jobs are processed in a queue and time required for completion of a given job is dependent on number of tasks pending in the server.

#### Server

**Figure 3:**
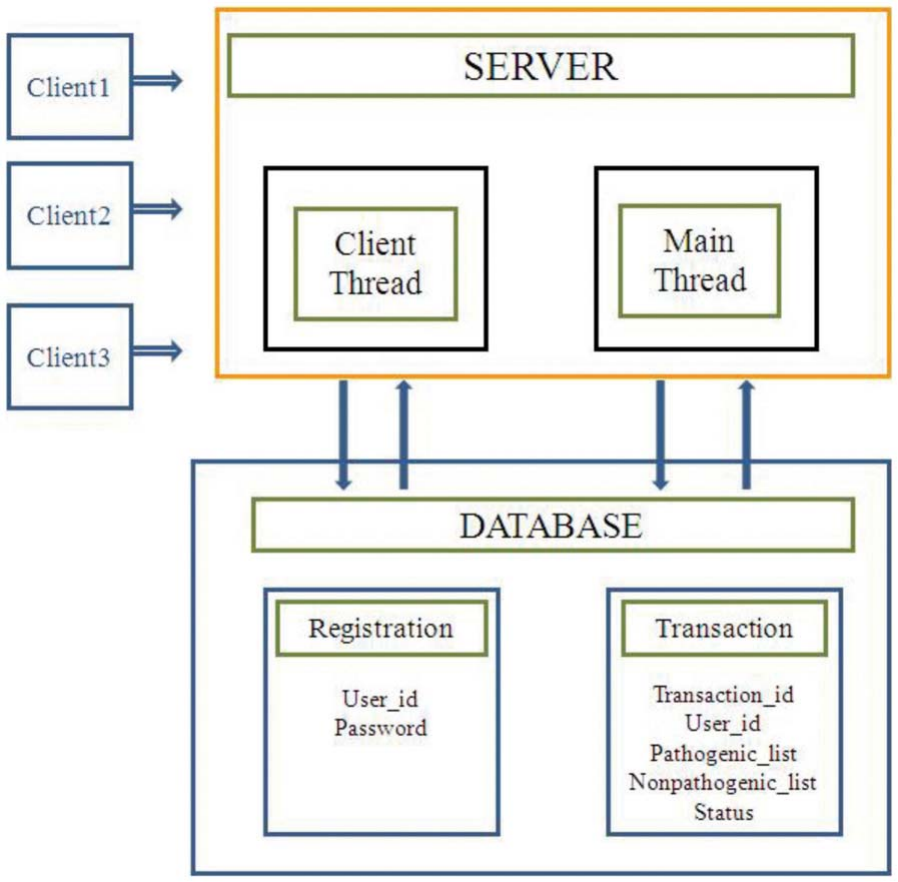
UniDrug-Target architecture.

The server was developed using CGI-Perl programming language by implementing multithreading and socket programming. The server has two threads interacting with a backend database developed on MySQL server. The backend database has ‘Transaction’ table having transaction_id, set_of_pathogenic strains, set_of_nonpathogenic strains, and status as its fields (Figure 3).

Once the server gets started, it initiates two threads: ‘client thread’ and ‘main thread’. The client thread waits until a socket gets connected, and once the socket gets connected, it takes pathogenic organisms list, nonpathogenic organisms list and inserts a new row into the transaction table for storing the respective fields information with status field information as ‘pending’ and waits for another socket to get connected. When main thread gets initiated, it extracts first pending transaction (job) in the transaction table and executes Perl scripts to implement the processes given in the flow diagram in Figure 2. Once a particular transaction is over, the results are generated and the server updates status field value transaction table to ‘Result’ from ‘pending’. Three hyperlinks (Complete HTML format, HTML/CSV format and Download HTML/CSV format) are generated on the place of later so that the user can retrieve results. The results will be stored at the site for a maximum time of one month. The main thread retrieves next pending transaction and repeats the above mentioned procedures.

### Data Representation

Once the user clicks on hyperlinks available in status column, the output is given in a table format with columns containing following information: 1. Serial No., 2. Highest matching non-pathogenic protein similarity score (in percentage), 3. GI number/synonymous name of the unique protein, 4. Essentiality of a unique protein or its similarity with the known essential proteins, 5. Protein function as a chokepoint or not along with its rank, 6. Whether unique protein is highly similar to known chokepoint enzymes in other pathogenic organisms along with rank(applicable for organisms in which chokepoint enzymes and ranks were not reported), 7. Hyperlink to the enzyme and its reactions data along with reactions uniquely consuming or producing metabolites with pathway connectivity graph starting from each reaction catalyzed by the enzyme (applicable when unique protein is an enzyme), 8. KEGG orthologous group ID, 9. Hyperlink to the pathways in which the protein is involved along with the pathway connectivity diagram, 10. Maximum similarity between unique protein and human proteome, 11. Domains and non-conserved residues information in pathogenic organism compared to non-pathogenic and 12. Unique active site information of pathogen protein sequences along with non-pathogen matching residue information.

### Implementation

The web server has been constructed by using CGI-Perl scripts with backend data developed from the available bacterial genome sequences and biological information represented in ‘data collection and generation’ section. Programs were developed for the identification of unique proteins at sequence level in association with standalone BLASTp when protein sequence of a pathogen fulfils two conditions one after another: sequence should be less than 30% similar (in entire length) with the proteins of beneficial bacteria or human and E-value greater than 0.0001. The output of two bacteria (*M. tuberculosis* H37Rv, *Bacillus anthracis* str. Ames) was checked manually and found that the above criteria were sufficient to identify pathogen-specific proteins in a proteome. These unique proteins were matched/compared with the database of essential bacterial proteins, enzyme sequences in the chokepoint database, data on proteins related to various pathways determined from orthologous genes involved in a pathway, and sequences of human proteome. Three new algorithms were developed and implemented into the server by using protein sequences, domains, structure, and metabolic reactions for; i. Construction of partial metabolic networks (PMNs) to determine extent of disturbances in metabolite production by targeting a protein; ii. Determination of conserved critical residues involved in protein-protein interactions, cavity formation and biological function at domain-level with low-matching sequences; and iii. Variation analysis of residues forming similar cavities between pathogenic, and nonpathogenic and human protein to identify cavities specific to pathogen. The server provides all the information mentioned in this section for each pathogen-specific protein in the order explained in ‘data representation’ section.

## Results

The web server, Unidrug-Target, has been designed to identify pathogen-specific unique drug targets in bacteria by indicating their pathogen specificity upto structural (cavity) and functional level. Data sets representing protein's functional importance and their potential to be considered as drug targets were generated by analyzing various available resources which were interconnected. Three new algorithms were developed and implemented as Perl scripts for PMNs construction to determine the affect of targeting a protein on metabolite disturbance in pathogen survival; domain level conservation among the low-matching pathogenic, non-pathogenic and human sequences to predict functional importance of low-matching regions and their role in representing critical residues such as surface residues involved in protein-protein interactions and cavity formation residues for protein-ligand interactions; and unique cavity identification in predicted unique proteins at sequence level to determine variability among the residues forming the same cavity in pathogenic and non-pathogenic organisms. The execution of this server is very simple and the user has to select pathogenic and non-pathogenic strains from the lists available at the server's website. The server provides list of unique proteins with their functional importance in an automated manner so that the user can make final decision on selection of a particular unique protein as a drug target.

The functional and other important features of unique proteins such as their essentiality in cell survival, chokepoint property, biological function, involvement in pathway(s) and inhibition of metabolite's production, residues conservation at domain level between proteins from non-pathogenic and pathogenic and residue sets forming unique composition at the cavities, which are specific to pathogen are provided by the server. The server also provides qualitative information regarding uniqueness of cavity at residue level for all unique proteins in the pathogens. Algorithms were also developed, implemented and incorporated into the server to provide choke point target efficiency, uniqueness at domain level and identification of cavity and surface residues of all predicted unique proteins. The relevance of developed algorithms in the prediction of unique proteins as drug targets is given below:

### Identification of chokepoint reaction efficiency of pathogen-specific proteins

Inhibition of unique proteins (enzymes) leads to interruption in consumption or production of a metabolite. Since no alternative enzyme or reaction in the bacterial system is available for chokepoint reaction(s), inhibition of unique chokepoint enzyme(s) involved in the consumption or production of a given metabolite may lead to accumulation or depletion of the metabolite in a cell which will be detrimental to the survival of pathogen. Analysis of disturbances in metabolite formation and pathways provides insight on potential of unique protein as a drug target. For example, K00189, KEGG orthologus group represents the enzyme, 3-Methyl-2-oxobutanoate dehydrogenase (EC: 1.2.7.7) which catalyzes the reaction involved in the production of 2-Methyl-propanoyl-CoA from 3-Methyl-2-oxobutanoic. The metabolite, 2-Methyl-propanoyl-CoA, is not produced by any other enzyme coordinated reactions or by any other pathway except valine, leucine and isoleucine degradation pathways. Starting from this reaction, the entire set of reactions in valine, leucine and isoleucine degradation pathway were traced upto the end metabolite, succinoyl-CoA, which is used as a substrate by 4 other metabolic pathways: citrate cycle, 1- and 2-methylnaphthalene degradation, benzoate degradation and reductive carboxylate cycle (involved in CO2 fixation). Benzoate degradation pathway further regulates several other pathways and the entire list of pathways under the influence of the enzyme can be seen Figure S2. This type of analysis is provided for all predicted proteins having annotated or highest possible match with the respective clusters of orthologous groups from the KEGG (KO groups). The drug target potential of each unique protein can be determined on the basis of relevance of these pathways in bacterium's pathogenesis and survival. Similarly, user can retrieve information on disturbances in pathways and metabolite production for any pathogen-specific enzyme of a bacterium by using the server. In standalone application, new pathways can be constructed, and even an entire new pathway connectivity diagrams can be reconstructed w.r.t. upcoming information.

### Identification of uniqueness in pathogen-specific proteins at the domain level

Similarity and dissimilarity among the residues in a common domain between the pathogenic and non-pathogenic protein sequences was analyzed to determine uniqueness in the pathogen-specific protein domains. This analysis would be useful in identifying conserved residues in low-matching regions because those residues might be important in preserving functionality which is otherwise difficult to predict directly from the comparative analysis between pathogenic and non-pathogenic sequences. This analysis is further extended to the identification of conserved residues involved in the cavity formation. For this purpose, the protein's domain sequence is mapped to available 3D protein structure so as to identify varying critical residues of interest involved in pocket formation between pathogen and non-pathogen sequences from low-matching regions. For example, one of the predicted targets, gi:15607938 in *M. tuberculosis*, matched with a region within Q743F5_MYCPA (Swissprot id) sequence containing Linocin_M18 (Pfam_id: PF04454.6) domain and the target showed low-matching with a set of sequences (GI: 108797777, 119866868, 126438337, 108802357, etc.) in the non-pathogenic organisms. The conservedness between pathogenic and non-pathogenic sequences w.r.t. their residue position matching with the domain sequence along with the role of matching position in pocket formation can be deciphered (Table 1).

**Table 1:** Sample analysis report of one of the pathogen-specific protein (gi:15607938) of *M. tuberculosis* to determine domain-level conserveness in low-matching sequences between pathogen-specific and non-pathogenic protein sequencesζ.

Pathogenic sequence:	F	E	G	Y	S	A	A	S	I	E	G	I	R	S	A	S	
15607938																	
Matching domain	F	E	G	Y	G	A	A	S	I	E	G	I	R	S	S	S	
sequence:Q743F5_MYCPA																	
Domain name:Linocin_M18																	
Conserved residues between	F	E	G	Y		A	A	S	I	E	G	I	R	S	+	S	
pathogenic and domain																	
sequenceφ																	
																	Matching
																	nonpathogenic
																	sequence_ids
		F	+	G	Y	+	A	A		+	E	G	I	+	A		108797777
		F	+	G	Y	+	A	A		+	E	G	I	+	A		119866868
		E						+		E		I					108802357
		E						+		E		I					119871510
		E						+		E		I					126438337
	F	E	G			A		I				+					108799508
	F	E	G			A		I				+					119868621
	F	E	G			A		I				+					108799508
3fdx_A			0	0	2		0	2	2		2	2		2	2		
Pocket numbersψ																	

ζ For detail see Algorithm section in ‘Material and Methods’.

φ Residue, +, blank and - indicate identity, similarity, dissimilarity and gap, respectively w.r.t. corresponding residues in pathogenic sequences in equivalent positions.

ψ Different residues forming a given pocket are represented by a number, i.e. 0 indicates biggest size pocket in a protein.

### Identification of unique cavity sites in pathogen-specific proteins

**Figure 4:**
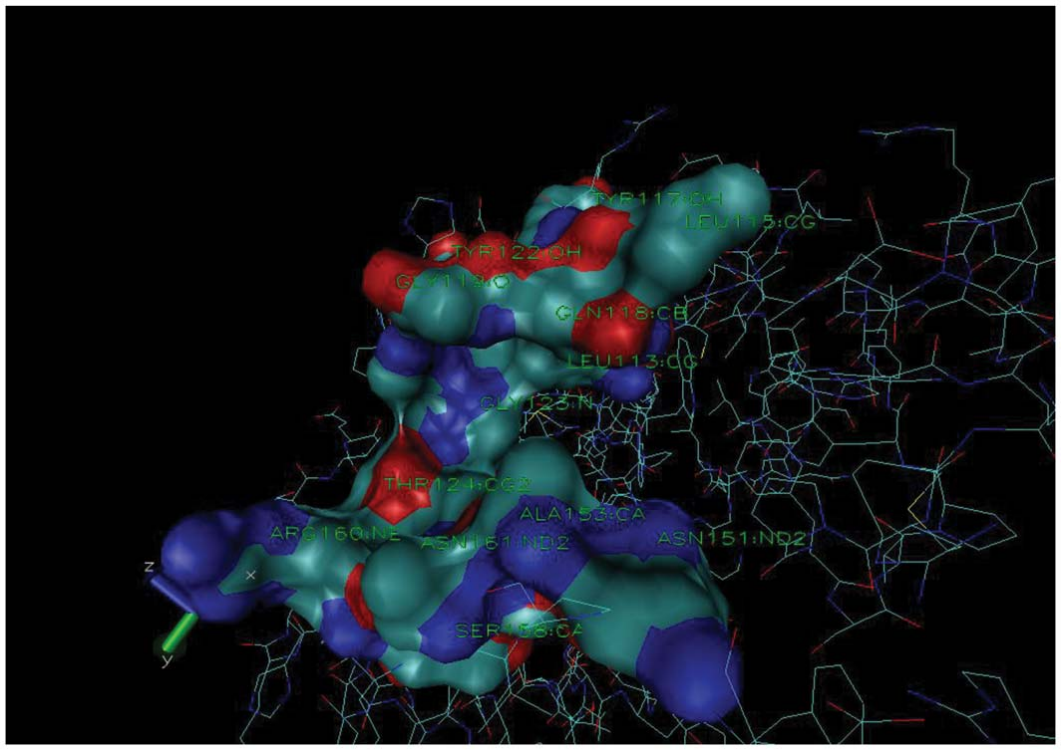
UniDrug-Target predicted pocket forming residues (**Table 2**) of gi:15607942 (PDBID: 2VZZ). Visualization through VMD (www.ks.uiuc.edu/Research/vmd/) showed that above residues forming a pocket. Pocket forming residues shown in the surface representation.

Uniqueness of cavities at residue level provides information related to the site to be targeted by drug molecules. Though this method requires user to analyze the output of the server manually to take a decision, still it provides possible residues in the cavities that are common among proteins of pathogen, non-pathogen and/or human, and also residues specific to the pathogen protein cavity. The tool can predict unique cavity information even for proteins for which 3D structures are not available by combining the information obtained from different low-matching known 3D PDB structures and their chains. For example, a pathogen-specific unique protein gi:15607942 with a known 3D structure (PDB ID: 2VZZ) [29] with three chains (A, B & C) is analyzed with low-matching 3D structure with PDB ID: 2VI7 to validate the algorithm designed for predicting possible pocket forming residues from the low-matching 3D structures for those protein sequences in which 3D structures are not available. The 3D structure of 2VZZ (gi:15607942) is 26% and 23% identical to 2VI7 chain C, and A & B, respectively. The residues forming pocket in 2VI7 structure are given in Table 2. From the matching data, corresponding pocket residues (refer to algorithms in materials and methods section) in 2VZZ, gi:15607942 are given in column 3 of Table 2. The predicted residues formed pocket in structure 2VZZ (Figure 4). These pocket forming residues were matched in non-pathogenic sequences to determine corresponding pocket forming residues and then unique or identical nature of the pocket in pathogenic is determined on the basis of dissimilarity or similarity of residues, respectively. Since equivalent pocket residues are different between the pathogenic and non-pathogenic sequences (Table 2), this pocket may have unique structure in the pathogen protein. The web server provides comparative analysis for each cavity present in each predicted unique sequence of pathogen by matching with available PDB structure information. The analysis will pinpoint cavities to be targeted within functionally important proteins so that the drug(s) developed against these pockets will not interact with the beneficial/non-pathogenic organisms and humans. A sample file for analysis is provided in tutorial at website of the server.

**Table 2:** Identified pocket residues of pathogen-specific protein, gi:15607942, matching with residues forming similar pocket in nonpathogenic sequences of different organisms *.

Residue position	Residue in pdb 2VI7	Residue in 2VZZ#		Nonpathogenic sequences residues (positions) along with match information							
in pdb 2VI7	(chain name)	& pathogenic sequence									
		15607942		126433780		126437231		108801252		108800463	
93	V(A)	L(113)	+	V(79)	+	V(121)	+	V(107)	+	P(141)	
94	A(A)	G(114)		D(80)		A(122)		A(108)		T(142)	
95	V(A)	L(115)	+	P(81)		S(123)		S(109)		V(143)	+
97	W(A)	Y(117)	+	Y(83)	Y	R(125)		R(11)		-	
98	Q(A)	Q(118)	Q	Q(84)	Q	I(126)		I(112)		R(144)	+
99	G(A)	G(119)	G	G(85)	G	G(127)	G	G(113)	G	G(145)	G
101	G(A)	G(121)	G	G(87)	G	G(129)	G	G(115)	G	G(147)	G
102	V(A)	Y(122)		I(88)		V(130)		V(116)		L(148)	
103	G(A)	G(123)	G	G(89)	G	A(131)		A(117)		G(149)	G
104	S(A)	T(124)	+	R(90)		T(132)	T	T(132)	T	Q(150)	
108	G(C)	A(128)		E(94)		A(136)	A	A(122)	A	R(154)	
111	L(C )	L(131)	L	L(97)	L	V(139)	+	V(125)	+	L(157)	L
112	D(C)	Y(132)		E(98)	+	D(140)	D	D(126)	D	D(158)	D
115	D(C)	F(135)		D(101)	D	F(143)	F	F(129)	F	D(161)	D
116	N(C)	A(136)		G(102)		G(144)		G(130)		A(162)	
131	N(A)	N(151)	N	N(113)	N	N(159)	N	N(159)	N	N(145)	N
133	P(A)	A(153)		P(115)	P	P(161)	P	A(147)		D(175)	
134	A(A)	S(154)	+	A(116)	A	S(162)	+	S(148)	+	N(176)	
136	A(A)	A(156)	A	A(118)	A	R(164)		R(150)		A(178)	A
137	L(A)	V(157)	+	R(90)		T(132)	+	T(118)	+	Q(150)	
138	Y(A)	S(158)		Y(120)	Y	L(161)		L(152)		Y(180)	
140	K(A)	R(160)	+	S(122)		K(168)	K	K(154)	K	R(182)	+
141	F(A)	N(161)		V(123)		V(169)		V(155)		F(183)	F

* The number indicates the gi: number. Residue, +, blank and - indicate identity, similarity, dissimilarity and gap, respectively w.r.t. corresponding residues in pathogenic sequences in equivalent positions.

# gi:15607942 is having an X-ray crystallographic structure with 2VZZ. The predicted residues are forming pocket equivalent to 2VI7.

## Discussion

The ‘UniDrug-Target’ web server is first of its kind and can identify potential pathogen-specific drug targets in bacteria. Drugs designed and used against those targets may cause few ecological problems and mitigate the problem of drug resistance. Bacterial strains with resistance to all but a single drug do exist, and some strains may soon have no effective treatments left in the “medicine chest”. The *Staphylococcus aureus* has developed resistance to penicillin, methicillin, tetracycline and erythromycin in a sequential order over the last fifty years and half of all its infections in the US were due to antibiotic resistant strains in 1991. This outcome left vancomycin as the only effective agent against the bacterium but surprisingly, strains resistant to this drug has also been identified in 2002 [30], [31]. Multidrug-resistant strains of *Enterococcus faecalis* and *E. faecium* have also shown resistances in the sequential order [32]. Penicillin and other beta-lactams resistances in *S. pneumoniae* are increasing worldwide and the major mechanism of resistance involves the occurence of mutations in genes encoding penicillin-binding proteins [33]. All strains of *M. tuberculosis* have shown resistance to known drugs. Recent studies have shown that few strains of *M. tuberculosis* have gained resistance to most effective drugs, isoniazid and rifampin [34]. Therefore, drug resistance emerged as a threat to established treatment.

Fast identification of effective and novel pathogen-specific drug targets is important in order to keep pace with the rapidly evolving drug resistance strains of bacterial pathogens. The web server, Unidrug-Target, has been designed to identify pathogen-specific proteins and their functional importance so that a user can make a decision regarding potentiality of a given protein to be used as a drug target. The predicted potential drug targets, through this server, will have the advantage of causing fewer adverse affects on humans as well as beneficial bacteria existing in the environment. As pathogenesis and survival processes differ among bacteria, this tool provides detailed information regarding unique proteins and leaves it to the user to take final decision on a given protein's drug target ability. The functional importance of each unique protein has been provided in an automated fashion by integrating diverse data: essentiality in cell survival, chokepoint property, biological function, involvement in pathways, pathways and metabolite's production getting disturbed by inhibiting unique proteins (enzyme cases only), sets of residues conserved at domain level between non-pathogenic and pathogenic organisms, and unique composition at the cavities specific to the pathogen by using known domain sequence and pocket information of available 3D structures in the PDB. The objective of the last parameter has been to provide unique cavities in a predicted protein target so that drugs designed against those cavities do not bind to any nonpathogenic organism. The computational method can point out residues which are surface exposed and forming unique cavity even for a protein without 3D structure.

UniDrug-Target is the first web server which provides potential pathogen-specific drug targets from proteome sequences based on their functional importance. Though many computational methods for identification of drug targets have been reported, but limitations in those computational methods impede their applications and usage. Li and Lai included only known human drug target sequences to train support vector machine (SVM) for the prediction of drug targets and, therefore, the method will unlikely predict unique drug targets in the bacterial pathogens [20]. Hopkins and Groom proposed ‘Lipinski rule-of-five’ (LR5) compliant molecule binding domain containing proteins which are not matching with any human sequences to be drug targets [15]. Hasan *et al.* applied the above principle to predict 354 drug targets in *M. tuberculosis* [16]. However, most of the proposed drug targets were present in many beneficial organisms [16]. Han *et al.* [21] used known targets from TTD database [35] to train SVM which predicts drug targets. Their method predicted large number of drug targets from a proteome (i.e. 845 drug targets out of 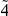
000 proteins of *M. tuberculosis*). Since most of the sequences used for training the SVM were from human and few from conserved proteins of bacteria, the predicted drug targets would show high similarity to the human and nonpathogenic bacterial sequences. Though their method was able to predict some commercialized and research targets in *M. tuberculosis* but from the predicted targets, most were having either similarity with the human proteins or non-pathogenic organisms.

Features such as pocket volume, accessible surface area, pocket compactness, ratio of individual residue frequencies on pocket surface to that of total surface, etc. are important in protein-protein interactions (PPIs). A SVM model containing 69 attribute features was earlier developed which included above mentioned pocket features to identify drug target potential of a protein from its 3D structure [36]. Critical residues forming similar pocket in non-homologous (low sequence similarity) or distinct protein structures has been identified using clique detection technique [37]. This finding supports our proposal of the use of low-matching 3D structures of proteins for the identification of plausible pocket forming residues in a pathogen-specific protein. However, the presence of a cavity in a target protein does not qualify it to be a druggable target because same cavity may be present in low-matching proteins of beneficial microbes or human proteomes. Therefore, identification of variation in residues forming cavity is an important parameter for emphasizing the protein as a druggable target as suggested in UniDrug-Target.

Drug target efficiency depends on functional role of the target in metabolic network of a pathogen, therefore, chokepoints have been proposed as possible drug targets. Rahman *et al.* proposed ranking of chokepoints based on number of shortest paths passing through the reaction catalyzed by the chokepoint enzymes between any two metabolites in the metabolic network of an organism and proposed highly ranked enzymes as better drug targets [27]. But it has been found that highly ranked enzymes are mostly housekeeping proteins and are also conserved across all organisms. Therefore, selecting those chokepoints as drug targets and developing drugs against those will lead to significant side-effects on humans and adverse impacts on the beneficial organisms. Kim *et al.* used simulated metabolic flux analysis to propose that a chokepoint reaction utilizing essential metabolite and inhibition of the enzyme (chokepoint) leading to zero mass growth in a bacterium to be used as a potential drug target [24]. As stated earlier, this approach is also expected to predict housekeeping proteins as drug targets. This method also lacked in representing the extent of impact of inhibition of chokepoint in a bacterium. In order to identify chokepoints as potential drug targets, those proteins should be specific to a pathogen and its potential in regulating metabolic network of bacteria should be identified. Through PMNs analysis, the server provides the pathways and end-metabolites to be disturbed by inhibiting a given unique enzyme. Further, none of the earlier computational drug target identification methods has mentioned the need to study proteins involvement in metabolic and signal transduction pathways to be used as drug targets. The UniDrug-Target provides the involvement of a given protein in different pathways so as to understand how a given drug disturbs different pathways.

UniDrug-Target server is expected to be useful to researchers not only for drug target identification but also for other purposes because the tool provides unique proteins as well as their functional information, which the biologists can use to identify the existence of unique biological processes specific to a pathogen or any bacterium that might be involved in virulence, pathogenesis, survival, persistence, etc. For example, when functionalities of predicted unique proteins of *M. tuberculosis* were analyzed, majority of the predicted proteins were found to be involved in persistence which is characterized largely as a slow metabolic process to overcome nutrient shortage and anaerobic conditions, changes in chemical environment surrounding the bacterium, and changes in morphology of cell wall surface of bacterium to protect it from immune responses. Interestingly, the identified unique proteins in *M. tuberculosis* were found to be involved in three important stages of persistence: initiation, continuation of persistence and reactivation to growth-phase to persistence. Similarly, unique proteins of any pathogen or a bacterium may be involved in any specific biological process which could be identified with the help of this server.

This server will add to the already known repertoire of drug targets in bacteria. In some cases, already known targets may be replaced with new targets and subsequently drugs can be developed against the new targets. When unique proteins of *M. tuberculosis* (determined by comparing proteome sequences of *M. tuberculosis* against (see systems and methods) beneficial *Mycobacteria* and human) were compared against proteomes of all beneficial bacteria, majority of the unique proteins (more 150 sequences) were identified to be having no or few sequence matches (1 or 2) with the sequences of all beneficial and non-pathogenic organisms. Even four unique proteins which are not matching with any of the beneficial organisms are found to be essential: gi:15609964 (a DNA binding protein implicated in the survival of *M. tuberculosis* in the macrophages of the host [38], gi:15610379 (hypothetical Protein), gi:15607884 (a DNA binding protein involved in transcriptional regulation), and gi:15607488 (conserved membrane protein). These proteins could be considered as new generation perspective drug targets against *M. tuberculosis*. Since the server is not pathogen-specific, it can provide potential drug targets for any pathogenic bacterium and expected to revolutionize pathogen-specific drug development.

## Supporting Information

Figure S1 Partial metabolic network (PMN) under the influence of pyrimidine metabolism pathway constructed by inhibiting the enzyme, dihydropyrimidine dehydrogenase.
(TIF)

Figure S2 Partial metabolic network (PMN) predicting the affect of inhibiting chokepoint reaction (R07160) involved in production of the metabolite, 2-Methylpropanoyl-CoA from 3-Methyl-2-oxobutanoic acid.
(TIF)
