# Supplementary figures and images for: UniDrug-Target: A Computational Tool to Identify Unique Drug Targets in Pathogenic Bacteria
Source: PLoS One. 2012 Mar 14;7(3):e32833. doi: 10.1371/journal.pone.0032833 (PMC3303792; doi:10.1371/journal.pone.0032833)

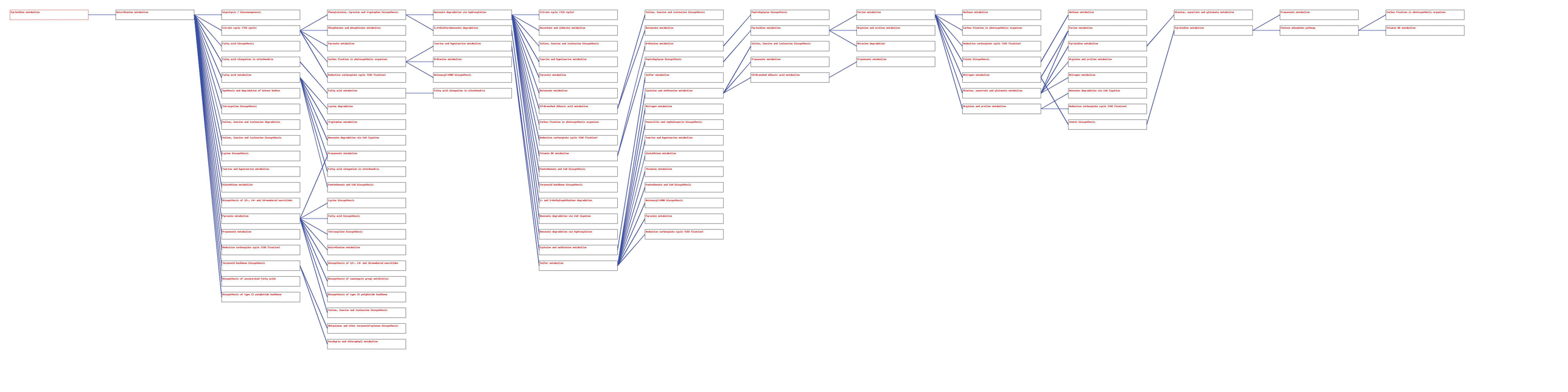

Supplement: Figure S1 — Partial metabolic network (PMN) under the influence of pyrimidine metabolism pathway constructed by inhibiting the enzyme, dihydropyrimidine dehydrogenase. (TIF) [file pone.0032833.s001.tif]

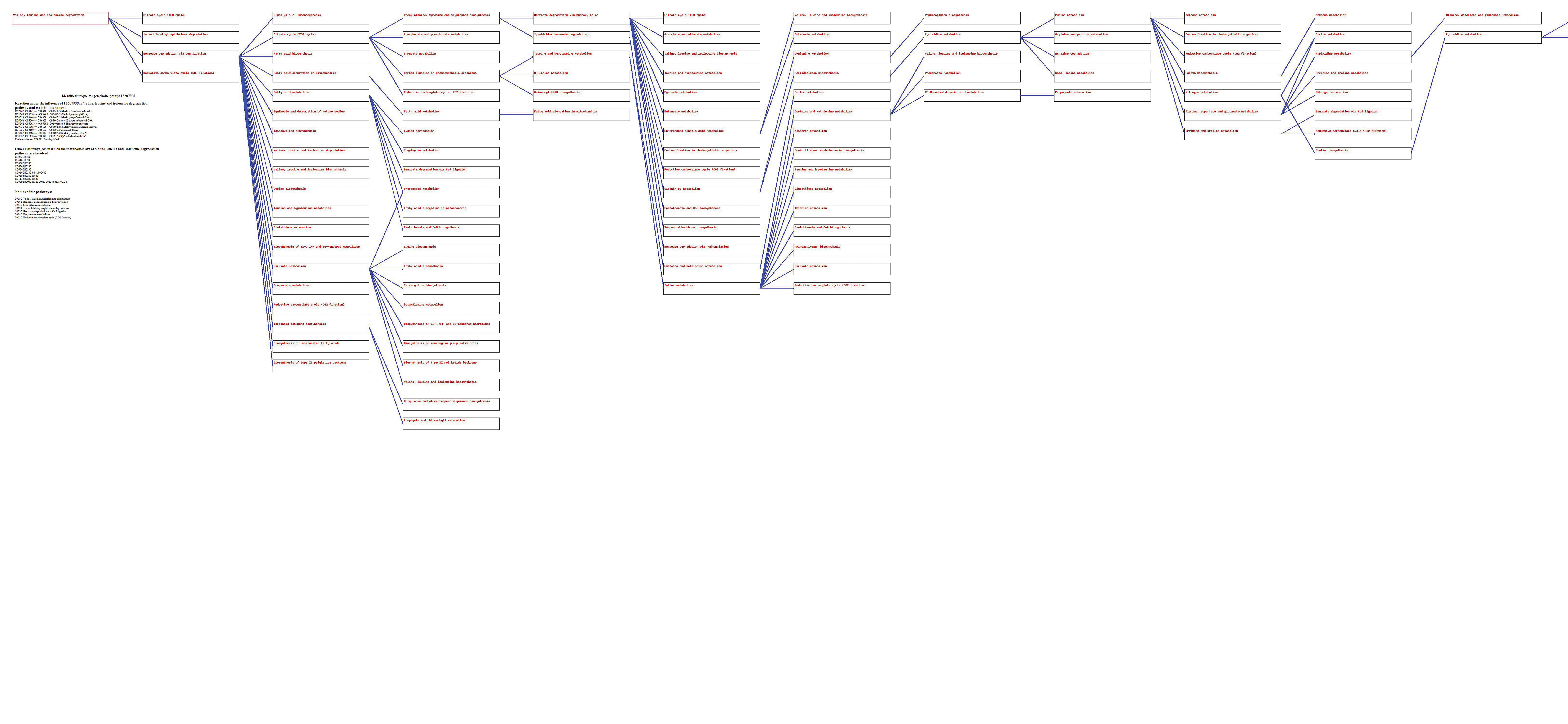

Supplement: Figure S2 — Partial metabolic network (PMN) predicting the affect of inhibiting chokepoint reaction (R07160) involved in production of the metabolite, 2-Methylpropanoyl-CoA from 3-Methyl-2-oxobutanoic acid. (TIF) [file pone.0032833.s002.tif]
